# Psychiatric Difficulties in Children with Celiac Disease and the Relationship between Adherence to Treatment and Parental Attitudes

**DOI:** 10.5152/tjg.2024.23493

**Published:** 2024-09-01

**Authors:** Didem Ayyıldız, Zeliha Demirtaş, Emine Kınacı

**Affiliations:** 1 Department of Child and Adolescent Psychiatry, Bursa Dörtçelik Pediatric Hospital, Bursa, Türkiye; 2 Department of Pediatric Gastroenterology and Hepatology, Bursa Dörtçelik Pediatric Hospital, Bursa, Türkiye; 3 Department of Pediatrics, Bursa Dörtçelik Pediatric Hospital, Bursa, Türkiye

**Keywords:** Celiac disease, child psychiatry, parenting, diet, gluten-free

## Abstract

**Background/Aims:**

Our knowledge of the factors related to parenting styles affecting adherence to diet in children with celiac diseases (CDs) and the association between psychiatric difficulties and diet compliance is largely based on limited data. Therefore, our work aims to examine primarily coexisting psychiatric difficulties in children with CD and raising attitudes of their parents and secondarily the relationship among adherence to treatment, psychiatric difficulties, and parental attitudes.

**Materials and Methods:**

Children aged 4–12 years (n = 42) who have been followed up with the diagnosis of CD in a Paediatric Gastroenterology Outpatient Clinic were compared with those of healthy controls (n = 31). One of the parents was asked to fill out the socio-demographic information form, Diet Compliance Form (only the patient group), “Parental Attitude Research Instrument” (PARI) and Strengths and Difficulties Questionnaire—parent form (SDQ).

**Results:**

The scores from “emotional problems,” “peer relationship problems,” and “total difficulties” areas were statistically significantly higher in the disease group than healthy controls. The average score of SDQ subscales and none of the PARI subscales differed between dietary compliance +/− groups. Significant positive correlations were detected between disease duration and PARI—overprotection/extreme motherhood (*r* = .421, *P* = .017) and PARI—strict/hard discipline (*r* = .368, *P* = .038) subscales.

**Conclusion:**

Clarifying the factors related to parenting that may affect patients’ adherence to a gluten-free diet will contribute positively to the course of the disease and the quality of life of patients and their families.

**Main Points**

- The association between psychiatric difficulties and diet compliance in pediatric celiac patients is largely based on limited data.
- Emotional problems and peer relationship problems subscales of the Strengths and Difficulties Questionnaire, indicating negative functionality, were higher in the disease group than in healthy controls.
- Significant positive correlations were detected between disease duration and Parental Attitude Research Instrument (PARI)—overprotection/extreme motherhood and PARI—strict/hard discipline subscales.
- The PARI subscales didn’t differ between dietary compliance +/- groups.

## Introduction

Celiac disease (CD), which is a common, chronic, autoimmune, inflammatory disease, is mostly treated with a gluten-free diet.^1^ CD prevalence has been found to be 0.47% in a population-based study conducted with school-age children across Türkiye.^2^ The chronic nature of the disease and the fact that the treatment is possible with a gluten-free diet adversely affect not only the child but also all family members.^3,4^ Previous research has shown that a deteriorated quality of life ^5-8^ and co-morbid psychiatric problems are common in children and adolescents diagnosed with CD and in their parents.^9^

Intestinal damage is known to be significantly associated with dietary compliance in CD patients.^10^ Previous studies have identified problems with accessibility to gluten-free foods as causes of non-adherence to dietary therapy.^11^ Comorbidity of psychiatric disorders, especially depressive symptoms, has also been known to impair adherence to gluten-free diet therapy.^12^ In addition to individual factors,^13^ such as coping skills, executive functions, or psychiatric problems, familial factors, particularly parental attitudes, are also effective in children’s treatment compliance.^14^

There have been several studies supporting the view that family functioning is an important indicator of quality of life and well-being in children and adolescents with chronic medical illnesses. Disruptions in family life were associated with unhealthier emotional/behavioral functioning and poor adherence to treatment.^15,16^ Studies in various chronic medical conditions point to the association between the style of parental attitudes and medical compliance, and the necessity to consider the family context during the treatment of the child.^17,18^

There is still a need for discussion on family-related factors that positively or negatively affect the treatment compliance of children and adolescents with CD. The aim of the research was thus to compare accompanying psychiatric difficulties in children with CD and the raising attitudes of their parents with those of healthy controls and to examine the relationship among adherence to treatment, psychiatric difficulties, and parental attitudes in the CD group.

## Materials and Methods

Children (aged 4 to 12 years), who have been followed up with the diagnosis of CD for at least 6 months in a pediatric gastroenterology outpatient clinic in Bursa Dörtçelik Pediatric Hospital, were retrospectively scanned, and their contact information was obtained. Children in a similar age range, who applied to the general pediatric polyclinic of the same hospital with acute upper respiratory tract complaints and did not have a chronic medical condition or psychiatric application, constituted the healthy control group.

This study was planned as a cross-sectional observational study. One of the parents was asked to fill out the socio-demographic information form, which includes the socio-demographic characteristics of the participants, and the Diet Compliance Form (only the patient group) to assess whether the child complied with the diet therapy and the difficulties they experienced in that regard. The “Parental Attitude Research Instrument” (PARI) and the Strengths and Difficulties Questionnaire-parent form (SDQ) were also given to the parents to fill out. The questionnaires prepared in the Google Forms application were delivered to the parents of the participants in the patient group via the WhatsApp application. The parents of the healthy controls were asked to fill out the scales during their outpatient clinic application. Necessary permissions for the study were obtained from Bursa Uludağ University Faculty of Medicine Clinical Research Ethics Committee (date: April 13, 2022 and number: 2022-8/2). The present study was conducted in compliance with the Declaration of Helsinki and informed consent was obtained from the participants.

### Measures

“PARI” was developed by Schaefer and Bell^19^ to assess parental attitudes. The Turkish validity and reliability study of the scale was conducted by Küçük^20^ The internal consistency coefficient of the scale was 0.91. The scale, which is made up of 5 different dimensions: Overprotection/extreme motherhood, Democratic approach/ensuring equality, Denial of housewife roles, Marriage conflict/disaccord, and Strict/hard discipline, has no total score. Scores from each sub-dimension were compared.

**The Strengths and Difficulties Questionnaire (SDQ)** is used to screen for emotional and behavioral problems. The scale has a form that parents of children aged 4-16 can fill out. The scale consists of 5 subscales: Behavioral Problems, Emotional Problems, Attention Deficit and Hyperactivity, Peer Relationships, and Pro-Social Behaviors. All except the pro-social subscale indicate negative functionality. The Turkish validity and reliability study was conducted by Yalın^21^ and it was found to be consistent and reliable in the Turkish sample.

**Diet Compliance Form: **An 8 item-questionnaire, which was created by the researchers of the thesis study titled “Investigation of Psychiatric Disorders and Quality of Life in Children and Adolescents Between 8-18 Years with Celiac Disease”, was used, after obtaining necessary permissions, to evaluate the treatment compliance. The scale consisted of questions about CD diagnosis duration, whether they had been hospitalized for CD, or complied with their diets. Parents were asked to respond with yes or no to the question, “Does your child comply with his/her diet completely?” In addition to the questions that parents could answer as yes/no, there were 2 open-ended questions about the difficulties they encountered while buying gluten-free foods or eating outside.^22^

### Statistical Analysis

The data were evaluated by using the Statistical Package for the Social Sciences (version 20.0) program (IBM Corp., Armonk, NY, USA). Descriptive statistics were shown as median, mean ± SD, or percentages (%). A 95% confidence interval was used to assess the data. Mann–Whitney *U*-test was used for comparisons between groups. Strengths and Difficulties Questionnaire (SDQ) total score variable was controlled by One-way Analysis of Covariance (ANCOVA). The correlations were tested by Spearman correlation analysis. For all analyses, statistical significance was set at *P *< .05.

## Results

Celiac disease group was consisted of 42 children with a mean age of (mean = 9.42, SD = 4.90), (median: 7.000); of these, 22 (52.4%) were girls and 20 (47.6%) were boys. The average age was (mean = 8.16, SD = 2.97), (median: 7.000) in the healthy control group (31), and of these, 18 (52.9%) were girls and 16 (47.1%) were boys. There was no statistically significant difference between the 2 groups in terms of age (* P* = 207, *F* = 23.124) and gender (*P* = 961).

It was found that the mean disease duration of the CD group was (mean = 3.36, SD = 1.1) years and just 20% of them had at least 1 hospitalization. Parents of children with CD, who agreed to participate in the study and completed filling out the scales properly, have reported that 28 (73.7%) of the participants adhered to their gluten-free diet. The answers given to the questionnaire “Diet Compliance form” revealed that the reasons for the difficulties faced by families when purchasing gluten-free products were high prices (87.5%), lack of gluten-free products/lack of knowledge of the artisans (75%), limited gluten-free product variety (12.5%), and the child’s craving gluten-containing foods (12.5%), respectively. According to the answers given for the other open-ended questions about challenges with eating out, 43.8% of the participants (14 of 32) stated limited options of eating places, 25% (8 of 32) uninformed managers, 12.5% (4 of 32) gluten cross-contamination, and 6.3% (2 of 32) high cost. Twenty percent of the parents (6 of 30) reported that they were unable to eat too, because their child couldn’t eat.

Further analyses showed that scores from “emotional problems,” “peer relationship problems,” and “total difficulties” areas were statistically significantly higher in the disease group than in healthy controls. The obtained score from the social skills sub-domain, where high scores indicate positive functioning, was higher in controls. There were no statistically significant differences between groups regarding PARI subscales. Comparative results showing psychiatric difficulty areas and PARI subscales are seen in Table 1.

When the disease group was divided into 2 groups based on treatment adherence, the mean scores of SDQ subscales and none of the PARI subscales differed between dietary compliance +/− groups according to the “Diet Compliance Form” (Table 2). When the SDQ—total score variable was controlled, the statistically insignificant findings on PARI subscales remained the same.

Correlation analyses indicated that there was a significantly negative association between age and SDQ—total score (*r* (42) = −.396, *P* = −.396, *P* = .001). The SDQ—total score was significantly correlated with only the PARI—democratic approach among PARI subscales, which is a positive parenting attitude. There was a negative, mild association (*r* (42) = −.279, *P* = .023). There was no significant relationship between average SDQ—total score and disease duration (*P* = .691). However, disease duration was found positively correlated exclusively with the SDQ—peer relations score among SDQ subscales (*r* (42) = .464, *P* = .003). Positive significant correlations have also been detected between disease duration and PARI—overprotection/extreme motherhood (*r* (42) = .421, *P* = .017) and PARI-strict/hard discipline (*r* (42) =.368, *P* = .038) subscales (Table 3).

## Discussion

The outcome of the present study reinforces the fact that children with CD have significantly higher levels of psychiatric difficulties in the emotional and peer relationship areas compared to healthy controls. Additionally, the majority of parents are having troubles with purchasing gluten-free products and eating outside due to high costs and a lack of knowledge among tradesmen.

Although coexisting psychiatric problems in CD have not been studied enough in children and adolescents, according to the results of a large-sample cohort study conducted with CD patients under the age of 18 in Sweden, the future risk of psychiatric disease in children with CD was found to be 1.4-fold higher than in the general population.^23^ According to the results of the study based on parent-reported mental health history of 73 celiac children in 2020, it was found that the rate of having at least 1 psychiatric diagnosis in children with celiac is higher than in the general population and anxiety disorders and ADHD have been detected as the most common diagnoses.^24^

There were conflicting results from studies conducted between 2014 and 2017 by a couple of different research groups from our country on comorbid psychiatric disorders in pediatric celiac patients. According to the results of the first one, which used semi-structured interviews, it was determined that 50% of the children had at least 1 psychiatric diagnosis.^25^ In Esenyel et al’s study, which used depression and anxiety scales and was conducted in the CD children with diet adherence at rates similar to ours, no difference was found in terms of psychiatric symptom levels compared to controls.^26^ According to the results of a third study with a small sample size in which “ The Depression Scale for Children” was used, it was stated that no significant difference was observed in terms of depression scores in children diagnosed with CD for whom diet therapy was recommended, and that the depression scores were significantly lower in the subgroup that adapted well to the diet.^27^ The small sample size of the studies and the fact that the age range in the latter 2 studies covers both the child and adolescent age groups make comparison difficult. According to the results of the former study in which psychiatric diagnoses were determined using K-SADS, conducted with children in the similar age range (8-12) as our study, the most frequently detected diagnoses—depression, anxiety disorders, and adjustment disorders—are compatible with our results, which detected increased difficulties only internalizing areas. In addition to parental interviews, reports from teachers of children in this age group have an important role in recognizing the symptoms of hyperactivity, attention deficit, and behavioral disorders. This might have led to the fact that there was no significant difference in terms of difficulty levels in the Hyperactivity-Inattention and Conduct problems areas of SDQ.

Studies investigating the deterioration in the quality of life of children with CD have been increasing in our country, and even a scale assessing the quality of life specific to the disease has recently been adapted into Turkish.^28^ In an up-to-date review article examining the quality of life and general well-being of children and adolescents with CD, it was concluded that they have been significantly negatively affected in terms of social functioning, although individual factors such as coping skills have a confounding effect.^29^ It is claimed that limitations in the gluten-free diet have an impact on children’s social activities.^30^ Therefore, our study results, which reveal that there is a moderately positive correlation between disease duration and only the peer relationships—psychiatric difficulty domain in celiac patients, corroborate the previous findings in the literature.

In addition to the fact that having a chronic disease poses a significant risk in terms of impaired quality of life and coexistent psychiatric problems in celiac patients, it might also be explained by the fact that the impaired gut microbiota is partially responsible for the immunological response to gluten, and that immunogenic peptides negatively affect brain functions.^31^ In a study conducted in 2004 on adolescents with CD using semi-structured diagnostic interview tools, major depressive disorder and disruptive behavior disorders were found to be significantly higher in CD compared to controls before the diagnostic biopsy, but no difference in after-biopsy psychiatric evaluation. It was suggested that these results may be related to the reduction of psychiatric symptoms in some patients after a gluten-free diet.^32^ Our findings would seem to demonstrate that there was no significant difference in terms of the psychiatric difficulties of children who were stated to comply with the diet and those who were not. It is plausible that the statistical analyses could have been influenced by the fact that the diet non-compliance group was so limited.

Adherence to a gluten-free diet has been linked to a better quality of life in a limited number of previous studies.^33^ Frequent follow-up visits, educational programs, and cooperation with a dietitian are among the factors that increase compliance. However, the factors regarding parents have become important considering the fact that control over the diet in childhood is provided mainly by the parents. Previous studies have shown that the level of parental knowledge and practice is determinant in terms of the child’s commitment to diet therapy.^34,35^ To the best of our knowledge, this report describes the first study that has examined the association between parental attitudes and diet compliance in children with CD. Contrary to expectations, we did not find significant differences in parenting attitudes based on diet compliance in the current study. This apparent lack of difference, emerging from data comparison, can be attributed to relatively short disease duration. The mean disease duration of the patients included in our study was 3 years, and the hospitalization history was present in just a small portion of them, which should be taken into consideration when evaluating the results. However, the most remarkable findings to emerge from our data are the moderate positive correlations between disease duration and both types of negative parenting style (overprotective and strict discipline) and the mild, negative correlation between the SDQ—total score and positive parenting attitude (PARI—democratic approaches subscale). It is known that parents’ own anxiety levels regarding children’s disease symptoms lead them to adopt protective attitudes. It might also be expected that parents of children with chronic diseases would use maladaptive coping skills more and exhibit negative parenting attitudes as their stress and depressive symptom levels increase. In addition, our results appear to be consistent with those of a recent study showing that the PARI—democratic subscale negatively predicted children’s emotional and behavioral problems, while the PARI—overprotective subscale positively predicted them.^36^

Our study has a number of weaknesses that need to be considered. Firstly, the fact that parental mental health, which might be a confounding factor, was not evaluated requires caution when interpreting the findings regarding parental attitudes. Secondly, the small sample size and the low number of cases not complying with the diet will negatively affect the generalizability of the statistical analysis results, which is another limitation that should also be taken into account. Another limitation of the study is that psychiatric diagnoses were not determined through psychiatric interviews. Finally, the parents of the control group filled out the forms in the outpatient clinic, while the parents of the disease group online. To minimize the negative impact of this limitation, scales that were wholly completed by both groups were included in the data.

Since the design of the previous studies was mainly cross-sectional, it is unclear whether low quality of life and psychiatric problems negatively affect compliance with the diet or vice versa.^37^ Longitudinal studies would contribute to understanding the direction of the relationship between the psychiatric challenges of celiac patients and diet compliance.

In conclusion, this study is the first step towards enhancing our understanding of the relationship between the psychiatric difficulties of children with CD and the attitudes of their parents. Studies with larger samples regarding the difficulties experienced by patients and their families in diet adherence and the familial factors related to dietary compliance would positively affect the course of the disease.

## Figures and Tables

**Table 1.** Descriptive Statistics Related to Psychiatric Difficulty Areas and Parental Attitude Dimensions

	CD (Mean ± SD)	Controls (Mean ± SD)	95% Confidence Interval of the Difference		*Z*	*P*
			Lower	Upper		
SDQ						
Conduct problems	1.90 ± 1.6	1.33 ± 1.2	−.13	1.27	−1.366	.172
Emotional problems	4.25 ± 2.8	2.09 ± 2.1	.95	3.36	−3.336	.001
Hyperactivity-inattention	4.35 ± 2.4	3.39 ± 1.6	−0.04	1.95	−1.637	.102
Peer relationship problems	3.40 ± 1.4	2.48 ± 1.4	.22	1.60	−2.699	.007
Pro-social behavior	8.30 ± 1.3	8.81 ± 1.4	−1.17	0.14	1.858	.063
Total difficulties score	13.90 ± 6.2	9.30 ± 4.0	2.07	7.11	−2.903	.004
PARI						
Overprotection/extreme motherhood	43.47 ± 9.0	44.18 ± 9.9	−5.34	3.92	.339	.734
Democratic approach/ensuring equality	27.00 ± 4.9	28.63 ± 3.4	−3.74	.46	1.247	.212
Denial of housewife roles	29.47 ± 6.7	28.45 ± 7.7	−2.52	4.55	−1.068	.286
Marriage conflict/disaccord	13.82 ± 4.2	14.33 ± 4.06	−2.54	1.52	.529	.597
Strict/hard discipline	39.41 ± 8.3	39.12 ± 9.2	−3.99	4.58	−.050	.960

CD, celiac disease; PARI, Parental Attitude Research Instrument; SDQ, Strengths and Difficulties Questionnaire;.

**Table 2.** SDQ and PARI Subscale Scores Based on Dietary Compliance

	Dietary Compliance (+) (Mean ± SD)	Dietary Compliance (−) (Mean ± SD)	95% Confidence Interval of the Difference		*z* (*P*) Unadjusted	*F* (*P*) Adjusted^a^
			Lower	Upper		
SDQ						
Conduct problems	1.92 ± 1.8	1.80 ± 1.2	−1.41	1.16	.841	
Emotional problems	4.21 ± 3.1	3.80 ± 1.6	−2.56	1.73	.698	
Hyperactivity-inattention	4.35 ± 2.6	4.60 ± 1.9	−1.62	2.10	.793	
Peer relationship problems	3.64 ± 1.0	3.00 ± 2.2	−1.71	0.43	.233	
Pro-social behavior	8.14 ± 1.3	8.80 ± 1.5	−.40	1.72	.218	
Total difficulties score	14.14 ± 6.8	13.20 ± 5.3	−5.80	3.91	.696	
PARI						
Overprotection/extreme motherhood	43.83 ± 10.0	40.25 ± 4.1	−11.13	3.96	.700 (.484)	.590 (.449)
Democratic approach/ensuring equality	27.58 ± 3.8	22.25 ± 7.9	−6.60	1.93	.352 (.725)	1.734 (.198)
Denial of housewife roles	29.75 ± 7.4	31.00 ± 2.0	−4.25	6.75	−.699 (.485)	.247 (.623)
Marriage conflict/disaccord	14.16 ± 4.5	14.50 ± 1.6	−3.06	3.72	−.176 (.861)	.445 (.510)
Strict/hard discipline	40.75 ± 9.4	35.25 ± 2.1−	−12.48	1.48	1.835 (.067)	1.706 (.202)

F, One-way analyses of covariance (ANCOVA); PARI, Parental Attitude Research Instrument; *z*, Mann–Whitney *U*-test.

^a^Adjusted for SDQ—total score.

**Table 3.** Findings of Correlation Analyses

	Disease Duration	SDQ—Total	PARI—Overprotection/Extreme Motherhood	PARI—Democratic Approach	PARI—Denial of Housewife Roles	PARI—Marriage Conflict	PARI—Strict Discipline
age	*P* = 175	*P* = **001**	*P* = 131	*P* = 051	*P* = 308	*P* = 674	*P* = 720
	*r* =. 225	*r* = −.396*	*r* = .191	*r* = .245	*r* = .129	*r* = .054	r = .046
Disease duration		*P* = 949	*P* = **017**	*P* = 149	*P* = 817	*P* = 706	*P* = **038**
		*r* = −.011	*r* = .421*	*r* = −.261	*r* = −.043	*r* = .069	r = .368*
SDQ—total			*P* = 377	*P* = **023**	*P* = 344	*P* = 251	*P* = 184
			*r* = .110	*r* = −.279*	*r* = .118	*r* = .143	*r* = .166
